# Alopecia and cutaneous atrophy due to occipital nerve block containing steroids

**DOI:** 10.1016/j.jdcr.2022.08.010

**Published:** 2022-08-10

**Authors:** Kaviyon Sadrolashrafi, Robert Lieberman, Narciss Mobini

**Affiliations:** a Department of Internal Medicine, Kirk Kerkorian School of Medicine at the University of Nevada, Las Vegas, Nevada; b Thomas Dermatology, Las Vegas, Nevada; c Associated Pathologists, Chartered/Quest Diagnostics, Las Vegas, Nevada

**Keywords:** alopecia, corticosteroid, cutaneous atrophy, occipital nerve block, scarring, steroid, H&E, Hematoxylin and eosin

## Introduction

Greater occipital nerve blockade is regularly used to treat primary headache disorders, including migraines and cluster headaches.^1^^,^^2^ Infiltration of the greater occipital nerve is particularly effective for patients with tenderness over the nerve.^3^ Corticosteroid use is not standard during these procedures; however, their use can induce prolonged relief.^1^ Various doses and preparations are employed in practice, often involving a combined injection of local anesthetics and corticosteroids around the occipital nerve.^1^^,^^2^ The cutaneous complication rate is higher with triamcinolone (lower solubility) than methylprednisolone or betamethasone (higher solubility).^4^^,^^5^ The risk of adverse cutaneous reactions also depends on the depth of the injection. More superficial subcutaneous or dermal injections carry significantly greater risk than deeper injections.^4^^,^^6^ The subject of this report is a rare case of alopecia developing after a nerve block procedure utilizing a solution of an anesthetic agent intermixed with a corticosteroid.

## Case report

**Fig 1:**
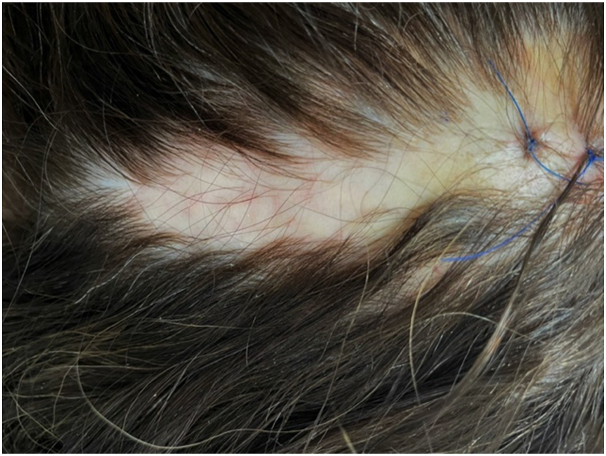
Irregular atrophic plaque of scarring alopecia in the mid-superior occipital scalp.

A 44-year-old woman presented for evaluation of a patch of hair loss she had noticed 2 months earlier. The patient’s past medical history included migraine headaches, and her social history included prior tobacco use. The patient denied recent changes to her hair care routine (ie, changing shampoos), using hair products such as straighteners or dyes, or applying excessive traction to the area. The patient reported undergoing an occipital nerve block 5 months ago to treat severe and intractable migraines. The injection site was separate from and inferior to the area of hair loss. Skin examination revealed a 4 × 3 × 2 cm vertically oriented, irregular plaque of alopecia accompanied by mild erythema, sclerosis, and atrophy (Fig 1). The affected area was tender to palpation.

**Fig 2:**
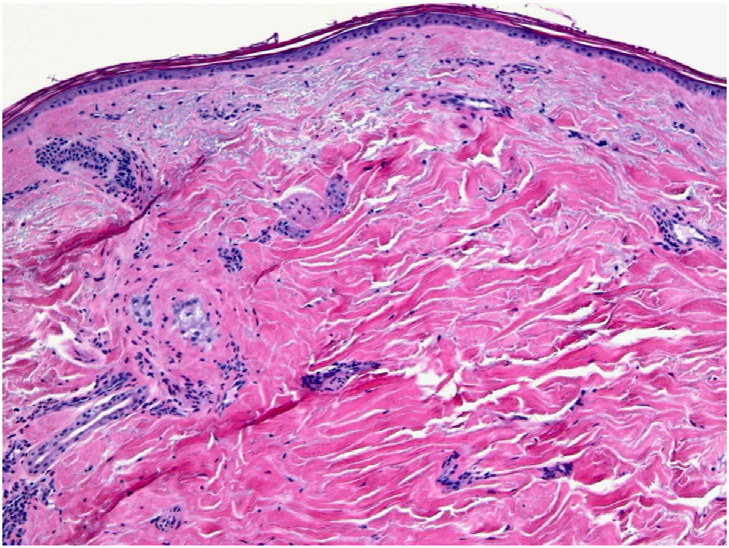
Epidermal atrophy, near loss of folliculosebaceous units, and dermal scar (skin punch biopsy. H&E stain; magnification: 10×).

**Fig 3:**
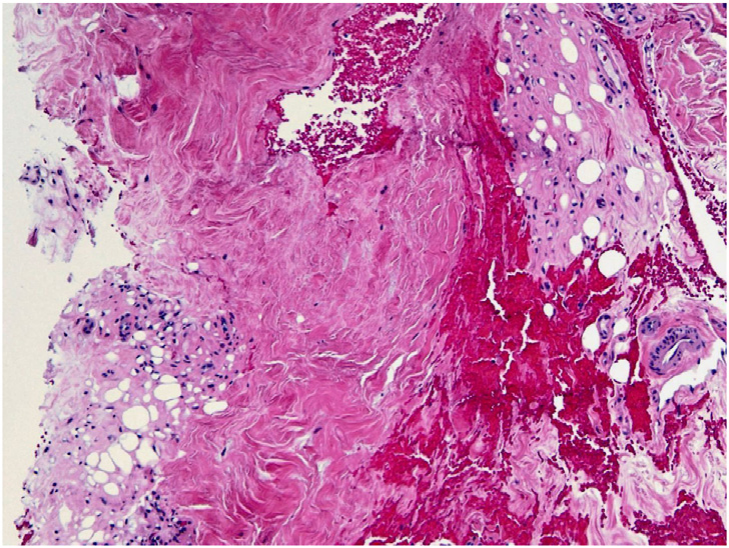
Dermal scarring and associated subcutaneous lipoatrophy (skin punch biopsy. H&E stain; magnification: 10×).

The patient’s clinical presentation was inconsistent with other common types of inflammatory scarring alopecias, such as discoid lupus erythematosus or lichen planopilaris. Our clinical differential diagnoses included morphea en coup de sabre, an atypical presentation of central centrifugal cicatricial alopecia (based on the shape of patchy hair loss), or sclerosis/scarring induced by a substance injected during the administration of the occipital nerve block. We performed a skin punch biopsy of the scalp for further evaluation. Histopathological examination revealed significant epidermal atrophy, focal dermal fibrosis with markedly diminished hair follicles, sebaceous gland atrophy, and underlying subcutaneous fat atrophy (Figs 2 and 3). Direct immunofluorescence studies were also performed, which failed to reveal any immunoreactant deposition.

These findings were most consistent with scarring alopecia and cutaneous atrophy, secondary to the effects of prior local corticosteroid use. Upon further investigation, we discovered that the solution injected during the nerve block was not a purely anesthetic agent and instead contained 1 mL bupivacaine 0.5% combined with 2 mL of methylprednisolone acetate (Depo-Medrol; 80 mg/mL). 1.5 mL of solution was injected bilaterally in the occipital head region with a 25-gauge needle around the points of maximal tenderness (ie, 2 injections were administered in total). The consent form provided during the procedure only detailed the potential risks of bleeding, infection, or nerve damage but did not mention any possible cutaneous side effects.

## Discussion

Corticosteroid use during nerve blocks is not commonplace.^1^ The steroid agent, if used, can induce cutaneous side effects. These reactions are rare, however, having been reported in less than 10 cases in published literature.^3^^,^^4^^,^^7^ Our report aims to familiarize dermatologists and dermatology practitioners with this rarely encountered but potentially significant side effect of nerve block procedures when dealing with unusual patterns of alopecia, especially when there is associated skin atrophy.

**Fig 4:**
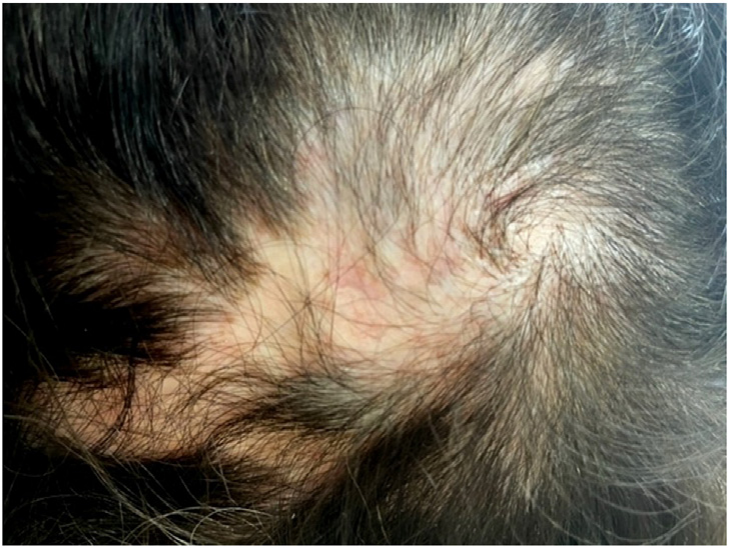
Gradual regrowth of fine hair, 8 months after the injection.

Induction of marked vasoconstriction of the dermal vasculature and the deposition of less soluble steroid crystals at the injection site appear to be mechanisms by which steroid injections produce skin atrophy and decrease the size of sebaceous glands and rate of hair growth. Steroid injections create an inhibitory effect on keratinocyte proliferation in the epidermis, leading to epidermal atrophy. These agents also inhibit type I and III collagen synthesis, fibroblasts, and the isoenzyme hyaluronan synthase 3. The reduction of hyaluronic acid within the extracellular matrix causes dermal atrophy.^8^^,^^9^ This condition demonstrates a variable course based on the extent of associated scarring. Resolution of cutaneous atrophy is reported to occur between 6 to 7 months postinjection but may persist up to 24 months.^3^^,^^4^^,^^8^^,^^9^ Fortunately, our patient began regrowing thin hair 8 months after her initial nerve block (Fig 4).

We hope our findings increase awareness of this rare and underreported complication from a commonly performed procedure. We also propose that consent forms of such procedures include this possible outcome in their documentation. Inclusion of this potential side effect is crucial for patient understanding and the establishment of informed consent. Female patients, in particular, may be more sensitive to an undesirable appearance of their scalp than male patients. Additionally, a subcutaneous depot injection may occur if component solubility varies and a heterogeneous combination forms between the 2 agents. If choosing to perform this procedure using a local anesthetic and corticosteroid, clinicians should only create a mixture of the solutions if they are miscible.^3^

## Conflicts of interest

None disclosed.
